# Modeling of Brain-Like Concept Coding with Adulthood Neurogenesis in the Dentate Gyrus

**DOI:** 10.1155/2019/2367075

**Published:** 2019-11-03

**Authors:** Ye Wang, Yan Gao, Yaling Deng, Lei Yang

**Affiliations:** ^1^Neuroscience and Intelligent Media Institute, Communication University of China, Beijing 100024, China; ^2^State Key Laboratory of Mathematical Engineering and Advanced Computing, Wuxi 214125, China; ^3^Pacific Northwest Research Institute, Seattle, WA 98122, USA

## Abstract

Mammalian brains respond to new concepts via a type of neural coding termed “concept coding.” During concept coding, the dentate gyrus (DG) plays a vital role in pattern separation and pattern integration of concepts because it is a brain region with substantial neurogenesis in adult mammals. Although concept coding properties of the brain have been extensively studied by experimental work, modeling of the process to guide both further experimental studies and applications such as natural language processing is scarce. To model brain-like concept coding, we built a spiking neural network inspired by adulthood neurogenesis in the DG. Our model suggests that neurogenesis may facilitate integration of closely related concepts and separation of less relevant concepts. Such pattern agrees with the previous experimental observations in classification tasks and place cells in the hippocampus. Therefore, our simulation provides insight for future experimental studies on the neural coding difference between perception and cognition. By presenting 14 contexts each containing 4 concepts to the network, we found that neural responses of the DG changed dynamically as the context repetition time increased and were eventually consistent with the category organization of humans. Thus, our work provides a new framework of word representation for the construction of brain-like knowledge map.

## 1. Introduction

Concept coding is a type of neural coding that abstracts perceptual information into cognitive concepts. The hippocampus is the core brain area responsible for concept coding [1, 2]. Neurophysiological experiments have shown that highly associated concepts share more hippocampal neurons, while less relevant concepts share fewer [3]. Neural coding of the learned concepts also changes dynamically during the learning process of new concepts [4]. The dentate gyrus (DG) is the first station for information processing in the hippocampus, which is the only brain region with adulthood neurogenesis besides the olfactory bulb [5]. The DG plays a vital role in regulating the neural coding distance between concepts, termed pattern separation and pattern integration [6]. Pattern separation is the ability of the brain to distinguish the difference between patterns to avoid confusion, while pattern integration contributes to encoding associative memories by reducing their neural coding distance. When the DG receives neural signals, the entorhinal cortex (EC) has already completed the abstraction and integration of multimodal perceptual information [7]. To further establish the concept level association between signals, the newborn neurons in the adulthood DG are shared by closely related concepts to decrease their coding distance, whereas it is more difficult for less relevant and temporally distant concepts to share newborn neurons, which increases their coding distance [8]. Thus, adulthood neurogenesis in the DG is a mechanism for adjusting the relationship between concepts dynamically [9]. Therefore, modeling brain-like concept coding by simulating the structure and function of the DG is essential for the construction of brain-like knowledge map.

Several simulation studies on the structure and function of the DG focused on pattern separation of the DG, but the phenomenon of pattern integration was mostly ignored. Myers and Scharfman [10] built a spiking neural network (SNN) with four major types of neurons in the DG: granule cell (GC), mossy cell (MC), hilar perforant path-associated cell (HIPP cell, HC), and basket cell (BC). They found that SNN can decrease the distance between concepts with shared neurons to achieve pattern separation. However, when HCs were lesioned, the activity of GCs increased and pattern separation improved, which appears to be inconsistent with the results of physiological experiments [11]. Faghihi and Moustafa [12] used the leaky integrate-and-fire model to build SNN to simulate the DG, and neurogenesis was added to both GCs and inhibitory neurons (including BCs and HCs). They found that pattern separation would be enhanced if the neurogenesis rate of GCs and inhibitory neurons was balanced. However, no evidence supported adulthood neurogenesis of inhibitory neurons. Chavlis et al. [13] used the multicompartment model to simulate GCs, and their network used signals from the EC as input to investigate the influence of neuronal morphology on pattern separation. They found that the sparseness of GC activity is crucial for pattern separation. Additionally, they found that the strengths of inhibitory connections, the properties of neurons such as leak conductance, size of soma, and number of dendrites could affect the sparseness of GC activity and thus pattern separation. From the perspective of pattern separation, the aforementioned models use too few concept categories (only two) and are difficult to generalize to guide applications. Furthermore, adulthood neurogenesis in the DG is the key biological process involved in both pattern separation and pattern integration [14, 15], but it was not incorporated in the aforementioned models except in Faghihi and Moustafa [12]. Therefore, models incorporating pattern integration and DG neurogenesis are needed to improve the simulation of brain-like concept coding in the DG. Aimone et al. [16] found that neurogenesis enhanced pattern separation for distal concepts; meanwhile, the coding similarity between concepts was increased when concepts were closer in time, which was the embodiment of pattern integration. However, when concept coding similarity in the EC was at 80%, pattern separation was still simulated; when the similarity of two visual stimuli is greater than 50%, they are likely classified as a single concept without pattern separation [17, 18]. Therefore, when concept coding similarity in the EC is at 80%, whether pattern separation occurs or not is still unclear. In addition, neurons in this network were simulated by the firing rate model with step size of 25 ms, but it is difficult to directly link such assumption to physiological data. Moreover, realistic time courses of synaptic interactions between neurons are ignored in Aimone et al. [16]. For these reasons, a direct dialog between models and cortical synaptic physiology is needed.

In this study, we propose a brain-like concept coding model based on SNN, taking adulthood neurogenesis into consideration, with subjectively generalized concept features as the evaluation criteria, and analyze the impact of input similarity, input time association, and neurogenesis rate on concept coding. This simulation of brain-like concept coding is the basis for the construction of brain-like knowledge map and enables a new level of artificial intelligence application.

## 2. Materials and Methods

### 2.1. Building the SNN

#### 2.1.1. Modeling Mature Neurons

Among the four main types of neurons (GC, MC, BC, and HC, refer to Introduction), GCs are the principle neurons, which are also the neurons with adulthood neurogenesis. MC, BC, and HC are three types of interneurons, among which MCs are excitatory neurons, and BCs and HCs are inhibitory neurons, which are used to regulate the neuronal activity of GCs. We used the adaptive exponential integrate-and-fire (AdEx) model, which reflects the behavior of four kinds of neurons in the DG, as the neuron model [13]. The AdEx model is

(1) $$\begin{array}{c} \begin{array}{c} C_{m}\frac{dV_{m}}{dt}=g_{l}\left(E_{l}-V_{m}\right)+g_{l}\Delta_{T}\exp\left(\frac{V_{m}-V_{T}}{\Delta_{T}}\right)+\sum^{}I_{\text{syn}}-w, \end{array}\\ \begin{array}{c} \tau_{w}\frac{dw}{dt}=\alpha \left(V_{m}-E_{l}\right)-w \end{array}, \end{array}$$

where *C*_*m*_ is the membrane capacitance, *V*_*m*_ is the membrane voltage, *g*_*l*_ is the “leak” conductance, *E*_*l*_ is the “leak” reversal potential (i.e., the resting potential), *I*_syn_ is the synaptic current flow onto the neuron, *w* is the adaptation variable, Δ_*T*_ is the slope factor, *V*_*T*_ is the effective threshold potential, *α* is the adaptive coupling parameter, and *τ*_*w*_ is the adaptation time constant. When the membrane potential reaches the firing threshold *V*_thr_, the neuron fires a spike, and then the membrane potential will be reset to a fixed value *V*_reset_, as follows:

(2) $$\begin{array}{c} \begin{array}{c} \text{when }V_{m}\geq V_{\text{thr}},\;V_{m}\;⟵\;V_{\text{reset}},\;w\;⟵\;w+b, \end{array} \end{array}$$

where  *b* is the spike-triggered adaptation parameter. The parameters of the neuron model [13] are shown in Table 1.

#### 2.1.2. Modeling Synapses

The DG network consists of both glutamatergic cells (GCs and MCs) and GABAergic interneurons (BCs and HCs); thus, both excitatory and inhibitory synapses are included in the network model. The excitatory and inhibitory postsynaptic currents are mediated by AMPA and GABA receptors, respectively. Both AMPA and GABA receptors are ligand-gated ion channels; the synaptic current can be described by

(3) $$\begin{array}{c} \begin{array}{c} I_{\text{syn}}\left(t\right)=g_{\text{syn}}\left(t\right)\left(V_{m}\left(t\right)-E_{\text{syn}}\right), \end{array} \end{array}$$

where *g*_syn_ and *E*_syn_ are the conductance and reversal potential of receptor, respectively. The reversal potential of AMPA and GABA receptors was set as *E*_AMPA_=0 mV and *E*_GABA_=−80 mV.

Once the receptor opens, the synaptic conductance first increases and then decreases over time; the dynamic model of receptor conductance is

(4) $$\begin{array}{c} \begin{array}{c} g_{\text{syn}}\left(t\right)=g_{\max}\exp\left(\frac{t-t_{s}+t_{\text{delay}}}{\tau_{\text{decay}}}\right), \end{array} \end{array}$$

where *g*_max_ is the maximum conductance, *t*_*s*_ is the spike time of preneuron, *t*_delay_ is the synaptic delay, and *τ*_decay_ is time decay constant. Simulations reported in this paper were all performed with *t*_delay_=2 ms and *τ*_decay_=6 ms.

#### 2.1.3. Plasticity Model

Spike timing-dependent plasticity (STDP) was observed in the perforant pathway between the EC and GCs [19]. Thus, we used STDP learning rule [20] as the plasticity model in our simulations; STDP is modeled as

(5) $$\begin{array}{c} \Delta w_{i}=\left\{\begin{array}{cc} A_{+}\exp\left(\frac{\Delta t_{i}}{\tau_{+}}\right), & \text{if }\Delta t_{i}<0,\\ A_{-}\exp\left(\frac{-\Delta t_{i}}{\tau_{-}}\right), & \text{if }\Delta t_{i}>0, \end{array}\right.\\ A_{+}=\left(w^{\max}-w_{i}\right)\eta_{+},\\ A_{-}=w_{i}\eta_{-}, \end{array}$$

where *η*_+_ and *η*_−_ are learning rates for potentiation and depression, *w*_*i*_ is the current synaptic weight, *w*^max^ is the maximum synaptic weight, Δ*t*_*i*_ is the delay time from presynaptic spike to postsynaptic spike, and *τ*_+_ and *τ*_−_ are delays which control the rates of exponential potentiation or decrease. Parameters were set as *w*^max^=2, *τ*_+_=20 ms, *τ*_−_=12 ms, *η*_+_=0.1, and *η*_−_=0.1.

#### 2.1.4. Modeling Developing Neurons

We focused on brain-like concept coding and assumed a simple form of newborn neuron maturation process, which was divided into five stages from birth to maturity. To encode more concepts in a short period of time, we simulated the accelerated maturation process of the newborn neurons within each stage for a few seconds, and the properties of developing neurons were modeled accordingly. Simulation of the accelerated maturation is a reasonable simplification of the model as the time scale of maturation and duration of concept stimuli representation are matched. To investigate the effects of time scales of neurogenesis on concept coding, we tested the maturation period of 5 s, 10 s, and 20 s in the simulation.

Since the newborn neurons in the DG are distinct from the mature ones, such as bearing stronger synaptic plasticity, lower inhibitory input, lower action potential threshold, higher resting state potential, and being easier to respond to novel stimuli [21–23], these developing neurons were simulated with the same model (Sections 2.1.1–2.1.3) but different parameters from the mature neurons in the network. To simulate stronger synaptic plasticity, lower action potential threshold, and higher resting membrane potential, we used extra current injection into the neurons under five stages corresponding to 100, 50, 20, 0, and 0 pA. To simulate lower inhibitory input, we multiplied the attenuation coefficient on the conductance of inhibitory synapses to GCs, with five stages corresponding to the values of 0.2, 0.6, 0.8, 1.0, and 1.0. To simulate ease to respond to novel stimuli, newborn neurons were modeled separately so that they are more likely to establish connections with current firing EC neurons. As the newborn neurons mature, the connection ratio of developing neurons to the current firing EC neurons gets smaller, which was set as 0.4, 0.3, 0.2, 0.1, and 0.1 in five stages. The parameters used in stage 4 and 5 are the same, which simulates the sustained mature state.

#### 2.1.5. Structure of SNN

**Figure 1:**
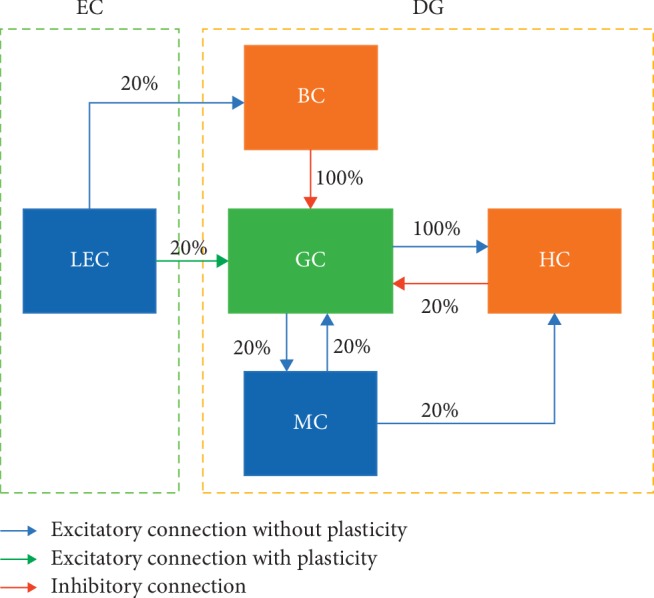
The structure of our simulated SNN. The LEC and DG are shown in hollow green and orange dotted boxes, respectively, the filled blue area represents excitatory neurons (LEC and MCs), the filled green area represents excitatory neurons with neurogenesis (GCs), and the filled orange area represents inhibitory neurons (BCs and HCs). The blue arrow represents excitatory connections without plasticity, the green arrow indicates excitatory connections with plasticity, and the red arrow represents inhibitory connections; the numbers near the arrows indicate the projection ratio.

The network (Figure 1) used in this study represents a local cortical circuit in the DG with input from the lateral EC (LEC); the LEC has been suggested to process the nonspatial contextual information needed to form episodic memories in the hippocampus [24]. For the sake of efficiency, the number of neurons in the network was scaled down corresponding to the actual number of neurons in the rat DG [25, 26]. Because neurons in the medial temporal lobe show binary coding to concept stimuli [9], the input neurons in the LEC were simulated as independent Poisson spike trains, with the frequency of 40 Hz. The simulated DG in our network included GC, MC, HC, and BC, and the projection ratios between layers are shown in Figure 1.

Because neural coding capacity is highly dependent on the number of neurons in the network [27]. We built three networks with an initial GC number of 200 (200 GCs), 400 (400 GCs), and 1,000 (1,000 GCs) to explore the performance of networks with different sizes on concept coding. The number of neurons in each layer and the neurogenesis rate of GCs are shown in Table 2. The parameters of the maximum synaptic conductance of networks with different sizes are shown in Table 3.

### 2.2. Generating Concept Stimuli

The LEC provides one of the two major input pathways to the hippocampus and was suggested to process the nonspatial contextual details of episodic memory [28]. Therefore, the neural coding of each concept in the LEC was used as the input of our model. The coding sparsity in the LEC was set to 0.1 [10, 13], i.e., each concept was represented with 10% active LEC neurons with a firing rate of 40 Hz, and the other LEC neurons fire spontaneously at a rate of 0.1 Hz.

The normalized dot product [16] was used to calculate the similarity between the cell layer outputs in response to two concepts, as follows:

(6) $$\begin{array}{c} \begin{array}{c} {\text{Sim}}_{A,B}=\frac{A\cdot B}{A\times B} \end{array}, \end{array}$$

where *A* or *B* is a 1 by *N* vector representing the firing rate of cell layer to concept *A* or *B*.

Then, we defined the pattern separation index (PSI) as

(7) $$\begin{array}{c} \text{PSI}=\left\{\begin{array}{cc} \frac{{\text{Sim}}_{\text{EC}}-{\text{Sim}}_{\text{GC}}}{{\text{Sim}}_{\text{EC}}},\;\; & \text{if }{\text{Sim}}_{\text{EC}}-{\text{Sim}}_{\text{GC}}>0,\\ \frac{{\text{Sim}}_{\text{EC}}-{\text{Sim}}_{\text{GC}}}{1-{\text{Sim}}_{\text{EC}}},\;\; & \text{if }{\text{Sim}}_{\text{EC}}-{\text{Sim}}_{\text{GC}}<0, \end{array}\right. \end{array}$$

where Sim_EC_ and Sim_GC_ indicate the similarity between concept pairs in the EC and GC layers. If PSI > 0, pattern separation occurs, and PSI is positively correlated with pattern separation effect. Otherwise, pattern integration occurs, and PSI is inversely correlated with pattern integration effect. Therefore, PSI can be used as a measurement of strength of pattern separation and pattern integration.

### 2.3. Experimental Setup

#### 2.3.1. Effects of Neurogenesis Rate, Input Temporal Association, and Input Similarity on Concept Coding

We used various neurogenesis rates, input temporal association, and input similarity on the simulated network to test the impact of these factors on concept coding. The experiment was separated into two phases: the training/growth phase and the test phase. During the training/growth phase, a pair of concept stimuli with coding similarity from 0% to 90% were presented to the network with each concept stimulus lasting for 1 s and a maturation period of 5 s. To investigate the effect of input temporal association on concept coding, we tested time intervals between two concepts ranging from 1 s to 5 s, where 1 s stands for presenting the next stimulus immediately after the previous one and 5 s stands for the next stimulus presented 5 s after the first stimulus onset, so that the time interval between two stimuli is inversely correlated with temporal association. We also simulated the performance of the model with maturation time of 10 s and 20 s, where the time interval between two concepts was proportionally increased to two and four folds to match the time scale of maturation time.

To explore the effects of neurogenesis rate on concept coding, we considered two situations: with and without neurogenesis. When a concept stimulus arrives, the GCs in the network experience neurogenesis according to the set neurogenesis rate. If there was no concept stimulus and thus no neurogenesis, the responses of the EC and GC layers were recorded after the training/growth phase, and the network was tested in each of the concept stimulus with plasticity and neurogenesis disabled.

For each neurogenesis rate, input temporal association, and input similarity, we ran the model 10 times from random seeds. The similarities between responses of the GCs to a pair of concept stimuli in these situations were then compared to the similarities between the neural coding of input EC neurons to two concept stimuli to get the PSI. We used analysis of variance (ANOVA) to assess the effects of neurogenesis rate, input temporal association, and input similarity on PSI and Bonferroni correction for the *post hoc* multiple comparison.

#### 2.3.2. Simulation of Brain-Like Concept Coding under a Variety of Contexts

The concepts are distributed representations consisting of semantic primitives or features. The overlap and differences in such feature-based representations can explain both the individuality of objects and their relationship to one another. Therefore, simulation of brain-like concept coding is the basis of brain-like knowledge map. We used the human-provided features of concepts as the evaluation criteria of brain-like concepts, which was obtained from a concept database (CSLB database) [29].

To explore the ability of the model on multiconcept coding, we showed 14 contexts each containing 4 concepts to the network with 200 GCs and a maturation period of 5 s, which is enough for coding the 56 concepts. The contexts and concepts can be found in [Supplementary-material supplementary-material-1]. The neural coding of each concept was generated randomly with similarity between any two concepts less than 50%. During the growth/training phase, the contexts were presented between 1 to 5 times to study the influences of repeated times on concept coding. Each concept was presented once during each context presentation, and the order of concept stimulus was random. During the test phase, all 56 concepts were presented to the network without neurogenesis and plasticity. For each context repetition time, we ran the model 10 times from different random seeds. We used ANOVA to assess the effects of context repetition time on the similarity between concepts in the cell layer and CSLB database and Bonferroni correction for the *post hoc* multiple comparison.

## 3. Results

### 3.1. Neurogenesis Rate, Input Temporal Association, and Input Similarity Have Significant Main Effects on Concept Coding

To study the impact of neurogenesis rate, input temporal association, and input similarity on concept coding, we tested these parameters on the 200 GC network with a maturation period of 5 s. We found significant main effects of neurogenesis rate (*F*_(5,2700)_ = 127.960, *p* < 0.001), input temporal association (*F*_(4,2700)_ = 312.478, *p* < 0.001), and input similarity (*F*_(9,2700)_ = 225.34, *p* < 0.001) on concept coding. The interaction effects between any two of the three factors were also significant (all *p* < 0.001).

**Figure 2:**
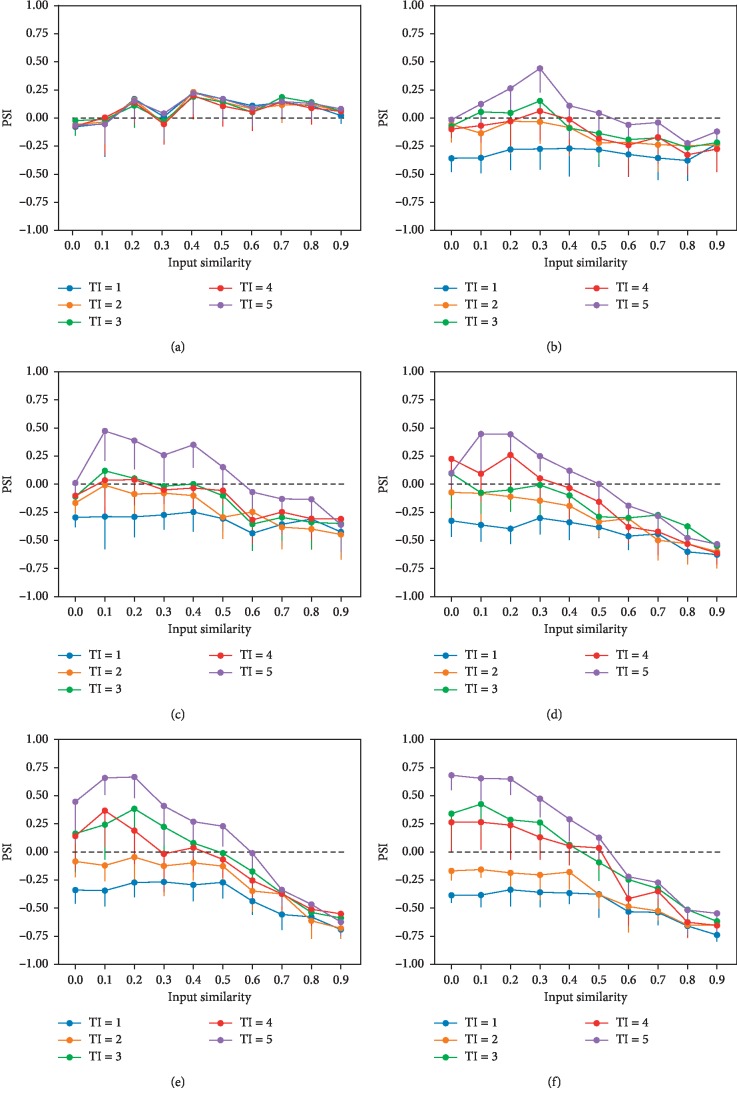
The impact of input similarity and input time interval on concept coding with the neurogenesis rate of (a) 0 neurons/s, (b) 1 neuron/s, (c) 2 neurons/s, (d) 3 neurons/s, (e) 4 neurons/s, and (f) 5 neurons/s in the 200 GCs network with a maturation period of 5 s where PSI > 0 means pattern separation, PSI < 0 indicates pattern integration, and PSI = 0 (black dotted line) presents neither pattern separation nor pattern integration. The error bars show standard deviation. NG: neurogenesis rate; TI: time interval.

Figure 2(a) shows the impact of input similarity and input time interval on concept coding without neurogenesis (neurogenesis rate of 0 neurons/s). As newborn neurons could not be shared by the presented concepts, the temporal association between the concepts did not affect PSI (*F*_(4,495)_ = 0.228, *p*=0.923). When input similarity is below 20%, a low level of pattern integration occurs (PSI = −0.044 ± 0.194), which may be induced by random connections between the EC layer and the GC layer; when input similarity is above 20%, weak pattern separation (PSI = 0.111 ± 0.175) occurs, which may be caused by sparse coding of the GCs [30].

Taking neurogenesis into consideration (neurogenesis rate above 0 neurons/s), time interval had a significant effect on PSI (all *p* < 0.001). When the neurogenesis rate is 1 neuron/s and input similarity is below 50%, the immature GCs shared between concepts induce pattern integration effect (PSI = −0.198 ± 0.228) if the two stimuli occurred within a short time of one another (within 1-2 s), while for events presented further apart in time, the influence of the immature GCs is reversed and pattern separation dominates (PSI = 0.033 ± 0.268). Nonetheless, if input similarity is above 50%, pattern integration occurs (PSI = −0.227 ± 0.222) no matter what the temporal association is, which may be induced by attractor effect [17, 18], as shown in Figure 2(b).

When the neurogenesis rate is between 2 neurons/s and 5 neurons/s and input similarity is below 50%, pattern integration dominates if the temporal association between two concept stimuli is strong; otherwise, pattern separation occurs. Meanwhile, if the input similarity is above 50%, pattern integration occurs regardless of the temporal association, which is consistent with results using neurogenesis rate of 1 neuron/s, as shown in Figures 2(c)–2(f).

**Figure 3:**
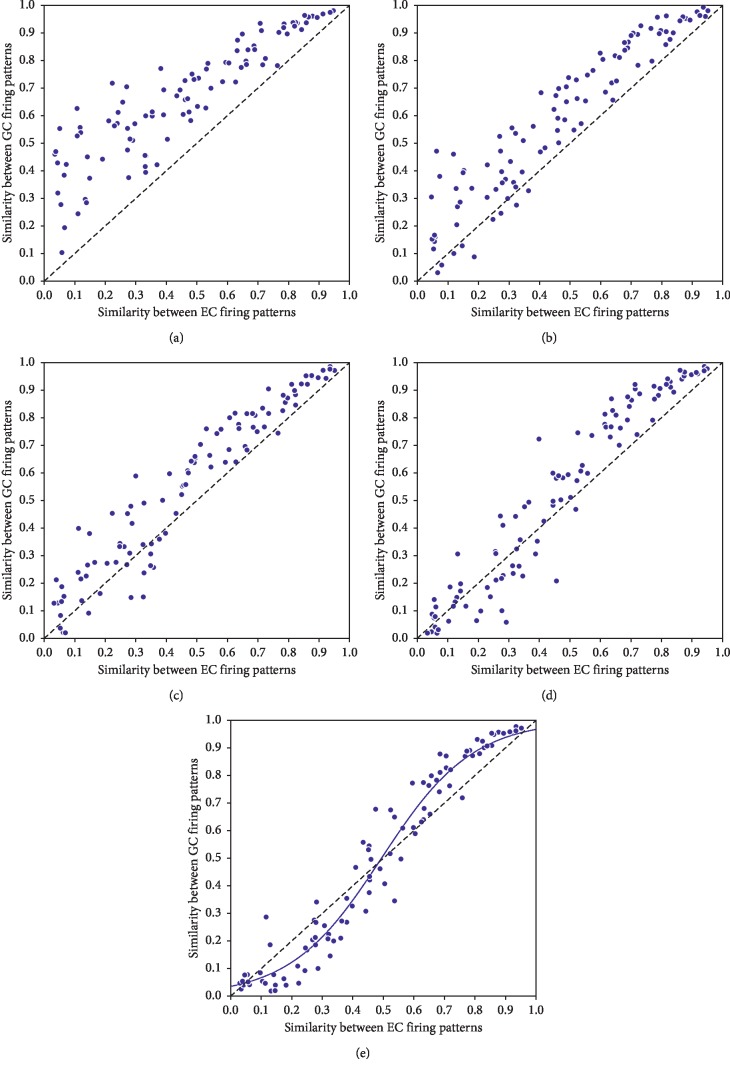
The scatter plots of the relationship between similarities of the firing patterns of the EC and the firing patterns of GCs with neurogenesis a rate of 3 neurons/s and an input time interval of (a) 1 s, (b) 2 s, (c) 3 s, (d) 4 s, and (e) 5 s in the 200 GCs network with a maturation period of 5 s. Each dot represents one concept pair stimuli with similarity between the firing patterns of the EC shown along the horizontal axis and similarity between the firing patterns of GCs shown along the vertical axis. Sigmoidal relationship can be found between similarities of the firing patterns of the EC and those of GCs with input time interval of 5 s. Black dashed lines denote the limit above which pattern separation is performed in the model. TI: time interval.

In addition, we investigated the impact of neurogenesis rate on PSI. Pattern separation or pattern integration effect is the strongest at the neurogenesis rate of 3 neurons/s (LSD correction, all *p* < 0.05). At this rate, the input time interval affects PSI significantly (*p* < 0.001), except for the time interval of 3 and 4 s (*p*=0.332)—the shorter the input time interval is, the easier it is to induce pattern integration. Figures 3(a)–3(d) show the relationship between similarities of the firing patterns of the EC and the firing patterns of GCs with a neurogenesis rate of 3 neurons/s and an input time interval of 1 to 4 s. Generally, the coding similarity in GCs is higher than that in the EC (black dotted line), which means that the shared developing neurons decreased the coding distance between concepts. As input time interval increases, pattern separation effect becomes stronger under low input similarity. However, when the time interval between concept stimuli is 5 s, no immature neuron could be shared by concepts and pattern integration still occurs under high input similarity. We fitted the relationship between similarities of firing patterns in the EC and GC layers with a neurogenesis rate of 3 neurons/s and an input time interval of 5 s with a sigmoid curve. When input similarity is below 50%, pattern separation occurs, and when it exceeds 50%, pattern integration dominates (Figure 3(e)).

### 3.2. Increased Maturation Time Does Not Affect Conclusions of the Simulations

We simulated the maturation process in the time scale of seconds, but mammalian neurons take weeks to mature [21]. Therefore, it is necessary to validate the observations in Section 3.1 with longer maturation period. We used two-fold (10 s) and four-fold (20 s) maturation periods of Section 3.1, and the duration of concept stimulus of 2 s and 4 s accordingly. By assessing the effects of neurogenesis rate, input temporal association, and input similarity on concept coding, we found significant main effects of neurogenesis rate (10 s maturation period: *F*_(5,2700)_ = 164.630, *p* < 0.001; 20 s maturation period: *F*_(5,2700)_ = 180.820, *p* < 0.001), input temporal association (10 s maturation period: *F*_(4,2700)_ = 432.971, *p* < 0.001; 20 s maturation period: *F*_(4,2700)_ = 458.248, *p* < 0.001), and input similarity (10 s maturation period: *F*_(9,2700)_ = 300.903, *p* < 0.001; 20 s maturation period: *F*_(9,2700)_ = 405.882, *p* < 0.001) on concept coding. The interaction effects between any two of the three factors were also significant (all *p* < 0.001), which is consistent with the conclusions of Section 3.1 using short maturation period.

The impact of input similarity and input time interval on concept coding with a variety of neurogenesis rates is demonstrated in [Supplementary-material supplementary-material-1] (200 GC network with a maturation period of 10 s) and [Supplementary-material supplementary-material-1] (200 GC network with a maturation period of 20 s), the result of which agrees with the maturation period of 5 s. At the neurogenesis rate of 0 neurons/s, the temporal association between the concepts had no effect on PSI (10 s maturation period: *F*_(4,495)_ = 0.592, *p*=0.669; 20 s maturation period: *F*_(4,495)_ = 0.753, *p*=0.556). At neurogenesis rates of above 0 neurons/s, the input time interval had a significant effect on PSI (all *p* < 0.001)—the weaker the temporal association is, the easier it is to induce pattern separation. The relationship between similarities of the firing patterns of the EC and those of GCs with the neurogenesis rate of 3 neurons/s is depicted in [Supplementary-material supplementary-material-1] (200 GC network with a maturation period of 10 s) and [Supplementary-material supplementary-material-1] (200 GC network with a maturation period of 20 s). These data suggest that neuron maturation time unlikely affects outputs of our simulations, and thus usage of 5 s neuron maturation time in the model is a reasonable simplification.

### 3.3. Increased Neural Network Scale Does Not Affect Conclusions of the Simulations

Biologically, the number of concepts that can be encoded is highly dependent on the neural network size, so we tested the properties of our simulated network with more neurons to find out whether network size affects the simulation. We repeated our simulation in the 400 GC and 1,000 GC networks. The highest neurogenesis rate for those simulations was proportionally increased to 10 neurons/s and 25 neurons/s, and the maturation period was set as 5 s. By examining the effects of neurogenesis rate, input temporal association, and input similarity on concept coding, we found significant main effects of neurogenesis rate (400 GCs: *F*_(5,2700)_ = 671.009, *p* < 0.001; 1,000 GCs: *F*_(5,2700)_ = 4146.271, *p* < 0.001), input temporal association (400 GCs: *F*_(4,2700)_ = 1117.016, *p* < 0.001; 1,000 GCs: *F*_(4,2700)_ = 2304.726, *p* < 0.001), and input similarity (400 GCs: *F*_(9,2700)_ = 1065.290, *p* < 0.001; 1,000 GCs: *F*_(9,2700)_ = 3086.902, *p* < 0.001) on concept coding. The interaction effects between any two of the three factors were also significant (all *p* < 0.001).

The impact of input similarity and input time interval on concept coding with a variety of neurogenesis rates is shown in [Supplementary-material supplementary-material-1] (400 GCs) and [Supplementary-material supplementary-material-1] (1,000 GCs). At the neurogenesis rate of 0 neurons/s, the temporal association between the concepts had no effects on PSI (400 GCs: *F*_(4,495)_ = 0.269, *p*=0.898; 1,000 GCs: *F*_(5,495)_ = 0.096, *p*=0.984). At neurogenesis rates of above 0 neurons/s, the input time interval had significant effect on PSI (all *p* < 0.001)—the shorter the input time interval is, the easier it is to induce pattern integration. The relationship between similarities of the firing patterns of the EC and those of GCs with a neurogenesis rate of 6 neurons/s in the 400 GC network and 10 neurons/s in the 1,000 GC network is depicted in [Supplementary-material supplementary-material-1] (400 GCs) and [Supplementary-material supplementary-material-1] (1,000 GCs). These data suggest that neural network scale unlikely affects conclusions drawn from our simulations.

### 3.4. Modeling of Brain-Like Concept Coding under a Variety of Contexts

To investigate the ability of our proposed model on multiconcept coding, we presented 14 contexts to the network one by one, each of which was presented for several times and then fed to the model to study the impact of repetition time on concept coding. The concepts were encoded in the GC layer with the neurogenesis rate of 3 neurons/s, which had the strongest effect on pattern integration and pattern separation (Section 3.1) and could ensure the balance between the numbers of excitatory (GCs and MCs) and inhibitory neurons (BCs and HCs) in the network.

**Figure 4:**
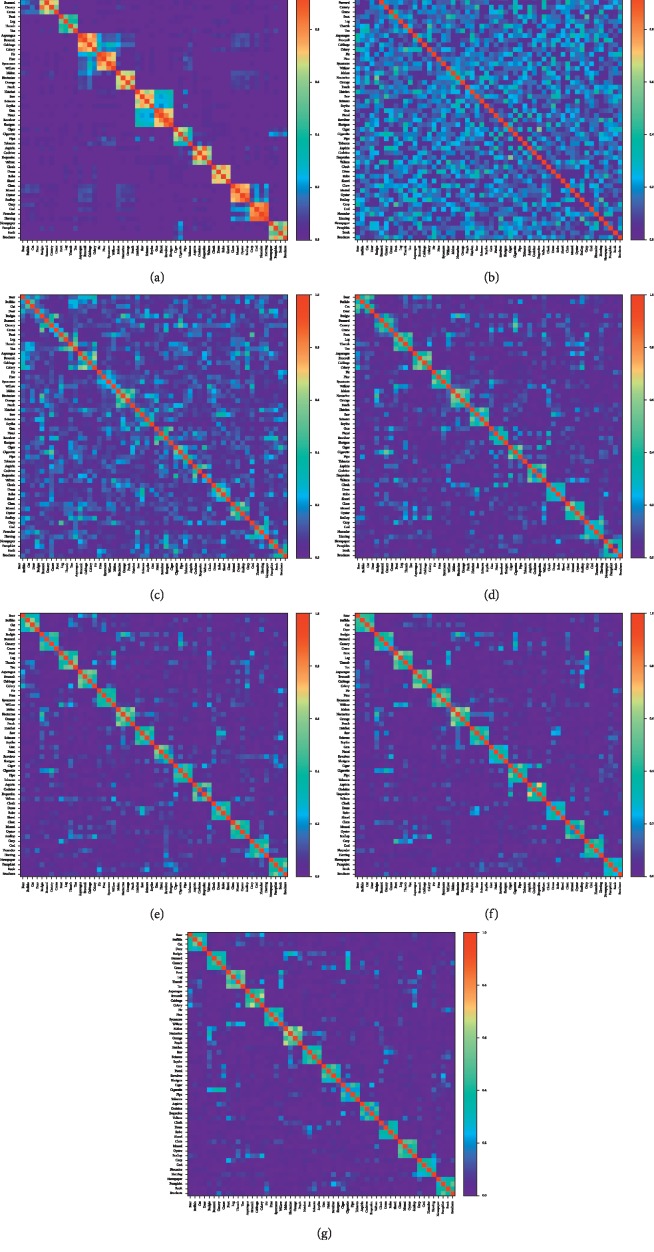
The concept similarity matrices of (a) the CSLB database, (b) the EC layer, and the GC layer with repetition times (rep) of (c) 1, (d) 2, (e) 3, (f) 4, and (g) 5.

Figure 4 shows the concept similarity matrices of CSLB database (Figure 4(a)), input neural coding in the EC layer (Figure 4(b)), and output responses in the GC layer with repetition time between 1 and 5 (Figures 4(c)–4(g)). From the matrices, we see that the representation of contexts significantly changed the coding similarity between concepts. The distance between concepts was random before experiencing the contexts (Figure 4(b)). After representation, the distance of concepts between the same context became smaller, whereas the distance between different context became larger (Figures 4(c)–4(g)), which agrees with pattern integration and pattern separation effect, respectively.

Furthermore, the similarity between concepts in the CSLB database and the proposed network was calculated as the normalized dot product between matrices (Table 4). We found that as repetition time of each context increased from 1 to 4, the similarity of coding distance in the GC layer and the CSLB database increased significantly (all *p* < 0.001), and no significant difference between repetition time of 4 and 5 was found (*p*=0.467). These data suggest that after the contexts are presented multiple times, the concept relationships became stable, and eventually, the similarity matrix of the GC layer became highly consistent with that of the CSLB.

## 4. Discussion

### 4.1. Insights for Neuroscience

In this study, we constructed a SNN with neurogenesis to investigate the comprehensive effects of neurogenesis rate, input time interval, and input similarity on concept coding in the DG. Our model suggests that neurogenesis may induce pattern integration for closely presented patterns. We observed attractor effect based on the sigmoidal relationship between input similarity in the EC and output similarity in the DG with weak temporal association. In addition, we found that intermediate level of neurogenesis (e.g., 3 neurons/s in 200 GCs) had the strongest effect on PSI, which may be a balanced state for developing neurons shared by concepts. The properties of the network were examined in a variety of maturation time scales and neuron numbers, and the conclusions are consistent among different conditions.

The results in our model suggest that among the situations with neurogenesis, when the temporal association between concepts is strong, pattern integration occurs because the concepts could share newborn neurons (Figures 2(b)–2(f)). Moreover, when the stimuli were presented far in time and the input similarity is below 50%, pattern separation was reduced without neurogenesis (Figure 3(e)). The role of adult-born neurons in the DG has been studied by neurogenesis knockdowns [15]: the adult-born GCs exhibited selectivity to single environment when rats experienced a long temporal separation between context exposures, and the selectivity was attenuated as the temporal separation between context exposures was shortened and the selectivity was further reduced with neurogenesis knockdown. This phenomenon is consistent with our model. Likewise, Danielson et al. [23] used the optogenetic technique to silence the adult-born neurons during exposure to the novel contexts, and they found impaired behavioral pattern separation in rats, which suggests the unique role of adult-born GCs in contextual discrimination behaviors. This is also consistent with our findings that neurogenesis could enhance pattern separation with large input temporal interval, where mature and adult-born neurons participate in coding familiar and novel contexts, respectively (Figure 2).

In our study, pattern separation dominates in the condition without neurogenesis (Figure 2(a)). Experimental studies also support such patterns: by analyzing the firing fields for the GCs of navigating rodents exploring morph environments, the DG was shown to disambiguate small differences in cortical input patterns [31]. This is consistent with our simulation results of no neurogenesis as the exploration of environments in the experiment was very fast (10 min) compared to the neuron maturation period. During spatial navigation, the DG receives highly specific spatial information from the medial EC (MEC) [32]. Thus, the results hint the possibility of altering our model to be used with the MEC as the input to study spatial coding.

In the longer temporal separation situation, we found that neurogenesis induces sigmoidal relationship between the input similarity and output similarity, which shows attractor effect (Figure 3(e)). A similar effect was also observed in behavioral studies to classify morphing cats and dogs [17]. Attractor effect was also found in place cells as the environment changes from square to circular gradually [18]. Currently, the relationship between perceptual input similarity and EC coding similarity is still unclear. Our simulation provides insight for future experimental studies to investigate the changing process of coding similarity from perception to cognition, especially in a variety of temporal associations.

### 4.2. Implications for Artificial Intelligence

By simulating the 14 brain-experienced contexts, our results suggest that during neurogenesis, the coding distance between concepts in the same context is decreased, and the coding distance between different scenes is increased (Figures 4(c)–4(g)). After pattern separation and pattern integration in the DG, the concept coding distance in the GC layer is highly consistent with the perception of the concepts using human subjects (Figure 4(a)). When each scene was presented 1 to 4 times, the similarity between concept relationship in the GC layer and the CSLB database reached 75.76%, 83.59%, 86.50%, and 87.69%, respectively, which means that the concepts within each context get closer as scenes are experienced for more times. Likewise, concept coding similarity is suggested to be positively correlated with association scores provided by the participants and web-based metrics [3]. Thus, the concept coding simulated in our model is comparable to the neural coding of human brains. Moreover, no significant difference was found between the 4th and 5th repetition (Figures 4(f) and 4(g)), which suggested that the relationship between concepts would be plateaued upon repetitive presentation of the same contexts. As a simulation of brain-like concept coding based on SNN, our work is the basis for the construction of brain-like conceptual knowledge map and provides the potential of achieving human-level artificial intelligence.

### 4.3. Limitations and Future Work

There are several limitations to our computational simulation. Firstly, the association between contexts may be heavily dependent on the pattern association function of the CA3 region of the brain, which contains large amounts of recurrent connections [33]. CA3 was not included in our model of the neural network, and thus the association between contexts with internal relations such as hardware tools and weapons would not manifest in this study. Therefore, CA3 is a candidate to be added to the neural network in the future work. Secondly, it has been shown that the neurogenesis rate in the DG is not constant—when we perceive novel stimuli, our brain will release dopamine, which in turn promotes neurogenesis [34–36]. Further studies are needed to establish the relationship between the novelty of concept stimuli and neurogenesis rate and to explore the effects of concept novelty on concept coding. Thirdly, cell death is an inevitable phenomenon in the brain [37], and increasing the number of GCs beyond certain threshold will lead to neural network imbalance. It is necessary for further studies to take cell death into consideration to model concept coding.

## 5. Conclusions

In this simulation study, we found that neurogenesis causes pattern integration for concepts with close temporal relationship and thus causes either pattern integration or pattern separation for concepts with far temporal relationship—depending on the similarity of the input concepts. The results were robust in a variety of maturation time scales and neural network scales. Furthermore, coding transformation is the most obvious with intermediate neurogenesis rate as opposed to extremely high or low rates, suggesting the existence of a balanced neurogenesis rate. By presenting 14 contexts of concepts to the network, we simulated dynamic neural coding of 56 concepts. As the repetition times of each context increases, the similarity between the neural concept coding matrix and the subjective concept coding matrix increases and eventually stabilizes, which provides a new framework of word representation for artificial intelligence. Our work serves as the foundation of potential future work to improve the model by adding CA3, concept novelty attribute, and cell death to the model, investigating the relationship between concept novelty and neurogenesis rate, establishing the association between contexts, and exploring the effects of neuron death on memory loss.

## Figures and Tables

**Table 1:** Model parameters for all neuron types.

Model parameters	GC	MC	BC	HC
*E*_*l*_ (mV) resting potential	−87.0	−64.0	−52.0	−59.0
*g*_*l*_ (nS) “leak” conductance	0.030	4.530	18.054	1.930
*C*_*m*_ (nF) membrane conductance	0.0067	0.6210	0.1793	0.0584
*V*_reset_ (mV) reset voltage	−74.0	−49.0	−45.0	−56.0
*V*_*T*_=*V*_thr_ (mV) threshold voltage	−56.0	−42.0	−39.0	−50.0
Δ_*T*_ (mV) slope voltage	0.0	2.0	2.0	2.0
*α* (nS) adaptation coupling parameter	2.0	2.0	0.1	0.82
*τ*_*w*_ (ms) adaptation time constant	45.0	180.0	100.0	93.0
*b* (pA) spike-triggered adaptation	45.0	82.9	20.5	15.0

**Table 2:** The number of neurons in each layer and the neurogenesis rate of GCs in the SNN with different sizes.

	LEC	GC (initial)	MC	BC	HC	NG (neurons/s)
200 GCs	100	200	50	25	50	0∼5
400 GCs	200	400	100	50	100	0∼10
1,000 GCs	500	1,000	250	125	250	0∼25

NG: neurogenesis rate.

**Table 3:** The maximum conductance of the SNN (unit: nS, each value represents the maximum conductance from neuron type of the column to neuron type of the row).

		GC	MC	BC	HC
200 GCs	LEC	10.0		12.0	
	GC		6.0		1.0
	MC	1.0			0.2
	BC	120.0			
	HC	20.0			

400 GCs	LEC	6.0		8.0	
	GC		10.0		0.4
	MC	1.0			0.1
	BC	120.0			
	HC	30.0			

1,000 GCs	LEC	3.1		5.0	
	GC		12.0		0.1
	MC	1.0			0.1
	BC	120.0			
	HC	20.0			

**Table 4:** The similarity (mean (%) ± std (%)) between coding matrices in the EC or the GC layer responses and the CSLB database under different repetition times (rep).

EC	GC (rep = 1)	GC (rep = 2)	GC (rep = 3)	GC (rep = 4)	GC (rep = 5)
56.59 ± 0.54	75.76 ± 1.17	83.59 ± 1.15	86.50 ± 1.08	87.69 ± 1.07	88.16 ± 0.73

## Data Availability

The data used to support the findings of this study are available from the corresponding author upon request.

## Supplementary Materials

Supplementary Materials Figure S1: the impact of input similarity and input time interval on concept coding with the neurogenesis rate of (A) 0 neurons/s, (B) 1 neuron/s, (C) 2 neurons/s, (D) 3 neurons/s, (E) 4 neurons/s, and (F) 5 neurons/s in the 200 GC network with a maturation period of 10 s, where PSI > 0 means pattern separation, PSI < 0 indicates pattern integration, and PSI = 0 (black dotted line) presents neither pattern separation nor pattern integration. The error bars show standard deviation. NG: neurogenesis rate; TI: time interval. Figure S2: the impact of input similarity and input time interval on concept coding with the neurogenesis rate of (A) 0 neurons/s, (B) 1 neuron/s, (C) 2 neurons/s, (D) 3 neurons/s, (E) 4 neurons/s, and (F) 5 neurons/s in the 200 GC network with a maturation period of 20 s, where PSI > 0 means pattern separation, PSI < 0 indicates pattern integration, and PSI = 0 (black dotted line) presents neither pattern separation nor pattern integration. The error bars show standard deviation. NG: neurogenesis rate; TI: time interval. Figure S3: the scatter plots of the relationship between similarities of the firing patterns of the EC and the firing patterns of GCs with a neurogenesis rate of 3 neurons/s and an input time interval of (A) 2 s, (B) 4 s, (C) 6 s, (D) 8 s, and (E) 10 s in the 200 GC network with a maturation period of 10 s. Each dot represents one concept pair stimuli with similarity between the firing patterns of the EC shown along the horizontal axis and similarity between the firing patterns of GCs shown along the vertical axis. Sigmoidal relationship can be found between similarities of the firing patterns of the EC and those of GCs with input time interval of 10 s. Black dashed lines denote the limit above which pattern separation is performed in the model. TI: time interval. Figure S4: the scatter plots of the relationship between similarities of the firing patterns of the EC and the firing patterns of GCs with a neurogenesis rate of 3 neurons/s and an input time interval of (A) 4 s, (B) 8 s, (C) 12 s, (D) 16 s, and (E) 20 s in the 200 GC network with a maturation period of 20 s. Each dot represents one concept pair stimuli with similarity between the firing patterns of the EC shown along the horizontal axis and similarity between the firing patterns of GCs shown along the vertical axis. Sigmoidal relationship can be found between similarities of the firing patterns of the EC and those of GCs with an input time interval of 20 s. Black dashed lines denote the limit above which pattern separation is performed in the model. TI: time interval. Figure S5: the impact of input similarity and input time interval on concept coding with the neurogenesis rate of (A) 0 neurons/s, (B) 2 neurons/s, (C) 4 neurons/s, (D) 6 neurons/s, (E) 8 neurons/s, and (F) 10 neurons/s in the 400 GC network with a maturation period of 5 s, where PSI > 0 means pattern separation, PSI < 0 indicates pattern integration, and PSI = 0 (black dotted line) presents neither pattern separation nor pattern integration. The error bars show standard deviation. NG: neurogenesis rate; TI: time interval. Figure S6: the impact of input similarity and input time interval on concept coding with the neurogenesis rate of (A) 0 neurons/s, (B) 5 neurons/s, (C) 10 neurons/s, (D) 15 neurons/s, (E) 20 neurons/s, and (F) 25 neurons/s in the 1,000 GC network with a maturation period of 5 s, where PSI > 0 means pattern separation, PSI < 0 indicates pattern integration, and PSI = 0 (black dotted line) presents neither pattern separation nor pattern integration. The error bars show standard deviation. NG: neurogenesis rate; TI: time interval. Figure S7: the scatter plots of the relationship between similarities of the firing patterns of the EC and the firing patterns of GCs with a neurogenesis rate of 4 neurons/s and an input time interval of (A) 1 s, (B) 2 s, (C) 3 s, (D) 4 s, and (E) 5 s in the 400 GC network with a maturation period of 5 s. Each dot represents one concept pair stimuli with similarity between the firing patterns of the EC shown along the horizontal axis and similarity between the firing patterns of GCs shown along the vertical axis. Sigmoidal relationship can be found between similarities of the firing patterns of the EC and those of GCs with input time interval of 5 s. Black dashed lines denote the limit above which pattern separation is performed in the model. TI: time interval. Figure S8: the scatter plots of the relationship between similarities of the firing patterns of the EC and the firing patterns of GCs with a neurogenesis rate of 10 neurons/s and an input time interval of (A) 1 s, (B) 2 s, (C) 3 s, (D) 4 s, and (E) 5 s in the 1,000 GC network with a maturation period of 5 s. Each dot represents one concept pair stimuli with similarity between the firing patterns of the EC shown along the horizontal axis and similarity between the firing patterns of GCs shown along the vertical axis. Sigmoidal relationship can be found between similarities of the firing patterns of the EC and those of GCs with an input time interval of 5 s. Black dashed lines denote the limit above which pattern separation is performed in the model. TI: time interval. Table S1: the contexts and concepts used in simulation.
