# Supplementary material for: Modeling of Brain-Like Concept Coding with Adulthood Neurogenesis in the Dentate Gyrus
Source: Comput Intell Neurosci. 2019 Nov 3;2019:2367075. doi: 10.1155/2019/2367075 (PMC6877936; doi:10.1155/2019/2367075)
Supplement: Supplementary Materials — Figure S1: the impact of input similarity and input time interval on concept coding with the neurogenesis rate of (A) 0 neurons/s, (B) 1 neuron/s, (C) 2 neurons/s, (D) 3 neurons/s, (E) 4 neurons/s, and (F) 5 neurons/s in the 200 GC network with a maturation period of 10 s, where PSI > 0 means pattern separation, PSI < 0 indicates pattern integration, and PSI = 0 (black dotted line) presents neither pattern separation nor pattern integration. The error bars show standard deviation. NG: neurogenesis rate; TI: time interval. Figure S2: the impact of input similarity and input time interval on concept coding with the neurogenesis rate of (A) 0 neurons/s, (B) 1 neuron/s, (C) 2 neurons/s, (D) 3 neurons/s, (E) 4 neurons/s, and (F) 5 neurons/s in the 200 GC network with a maturation period of 20 s, where PSI > 0 means pattern separation, PSI < 0 indicates pattern integration, and PSI = 0 (black dotted line) presents neither pattern separation nor pattern integration. The error bars show standard deviation. NG: neurogenesis rate; TI: time interval. Figure S3: the scatter plots of the relationship between similarities of the firing patterns of the EC and the firing patterns of GCs with a neurogenesis rate of 3 neurons/s and an input time interval of (A) 2 s, (B) 4 s, (C) 6 s, (D) 8 s, and (E) 10 s in the 200 GC network with a maturation period of 10 s. Each dot represents one concept pair stimuli with similarity between the firing patterns of the EC shown along the horizontal axis and similarity between the firing patterns of GCs shown along the vertical axis. Sigmoidal relationship can be found between similarities of the firing patterns of the EC and those of GCs with input time interval of 10 s. Black dashed lines denote the limit above which pattern separation is performed in the model. TI: time interval. Figure S4: the scatter plots of the relationship between similarities of the firing patterns of the EC and the firing patterns of GCs with a neurogenesis rate of 3 neuro [file 2367075.f1.docx]

# Supplementary Figures


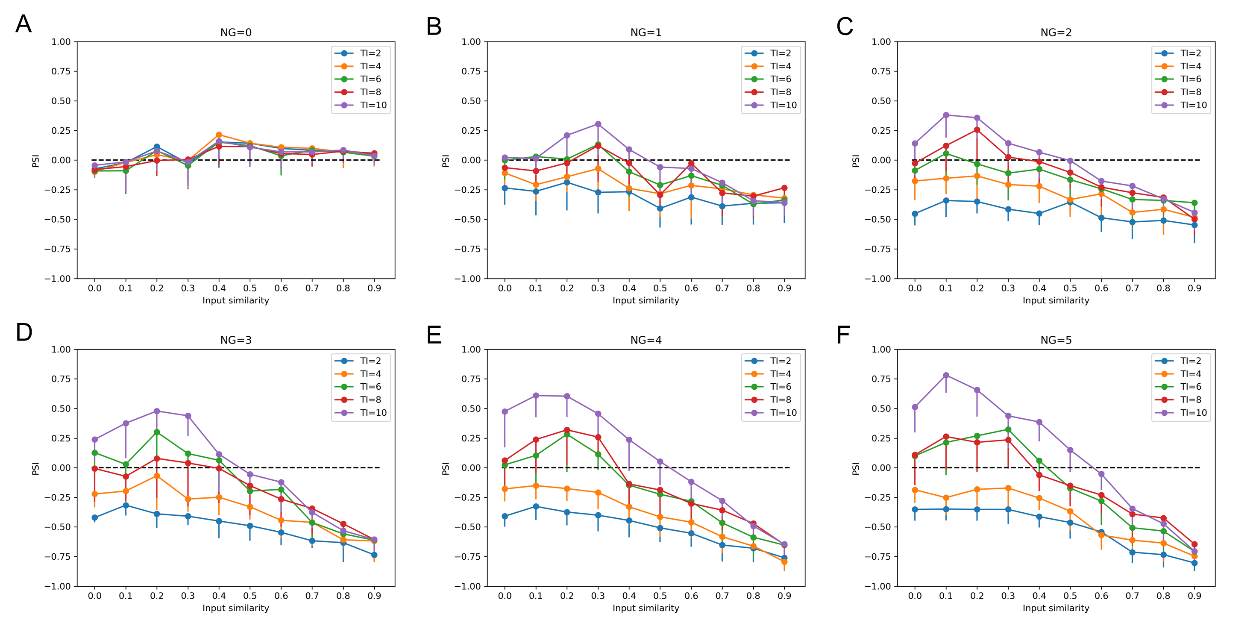


Figure S1. The impact of input similarity and input time interval on concept coding with the neurogenesis rate of (A) 0 neuron/s, (B) 1 neuron/s, (C) 2 neurons/s, (D) 3 neurons/s, (E) 4 neurons/s and (F) 5 neurons/s in the 200 GCs network with maturation period of 10 s, where PSI>0 means pattern separation, PSI<0 indicates pattern integration, and PSI=0 (black dotted line) presents neither pattern separation nor pattern integration. The error bars show standard deviation. NG: neurogenesis rate, TI: time interval.


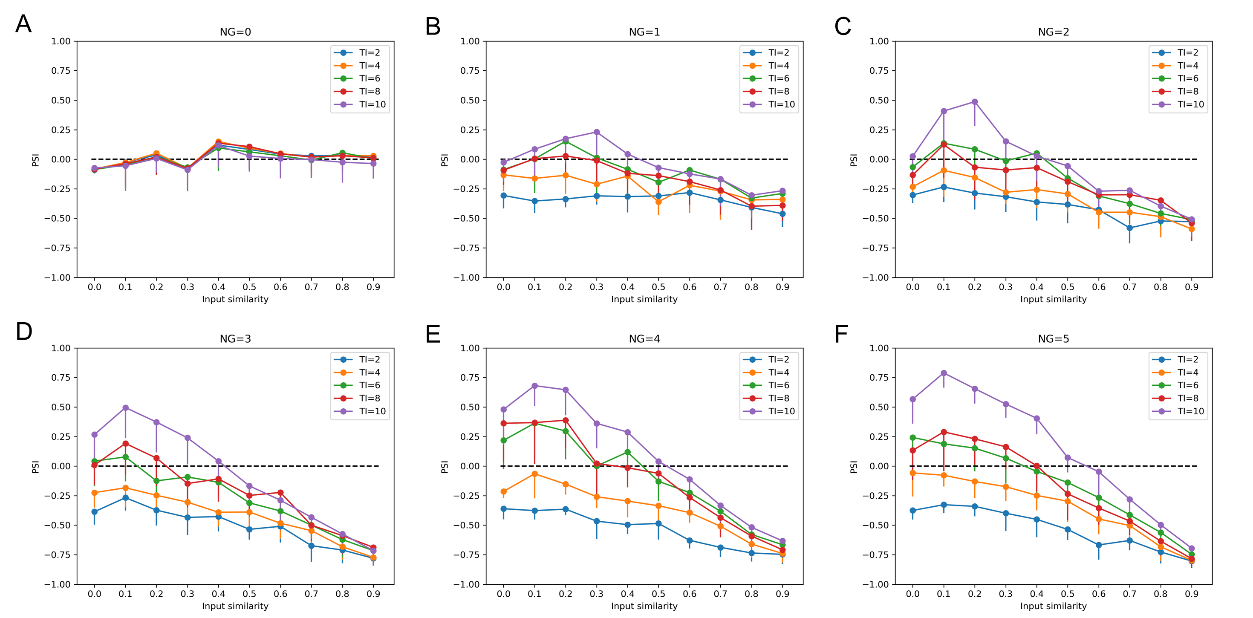


Figure S2. The impact of input similarity and input time interval on concept coding with the neurogenesis rate of (A) 0 neuron/s, (B) 1 neuron/s, (C) 2 neurons/s, (D) 3 neurons/s, (E) 4 neurons/s and (F) 5 neurons/s in the 200 GCs network with maturation period of 20 s, where PSI>0 means pattern separation, PSI<0 indicates pattern integration, and PSI=0 (black dotted line) presents neither pattern separation nor pattern integration. The error bars show standard deviation. NG: neurogenesis rate, TI: time interval.


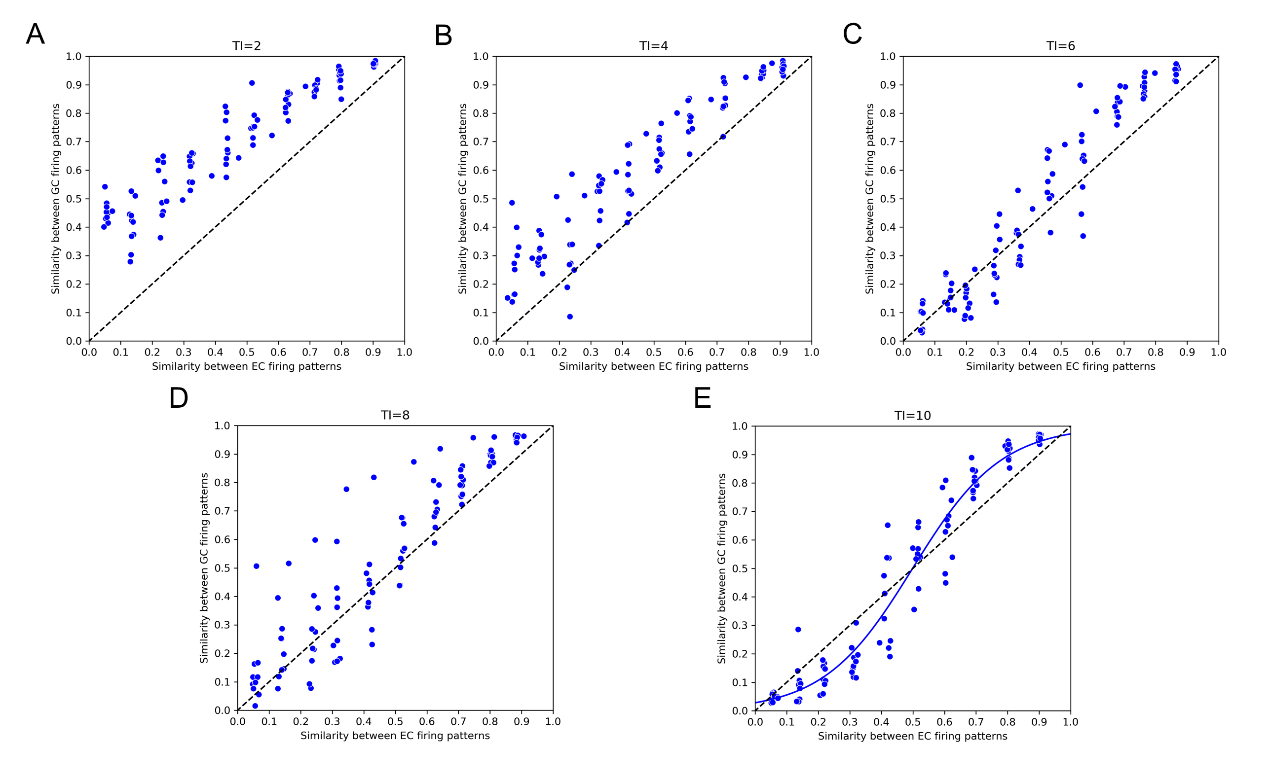


Figure S3. The scatter plots of the relationship between similarity of the firing patterns of EC and the firing patterns of GCs with neurogenesis rate of 3 neurons/s and input time interval of (A) 2 s, (B) 4 s, (C) 6 s, (D) 8 s and (E) 10 s in 200 GCs network with maturation period of 10 s. Each dot represents one concept pair stimuli with similarity between the firing patterns of EC shown along the horizontal-axis and similarity between the firing patterns of GCs shown along the vertical-axis. Sigmoidal relationship can be found between similarity of the firing patterns of EC and that of GCs with input time interval of 10 s. Black dashed lines denote the limit above which pattern separation is performed in the model. TI: time interval.


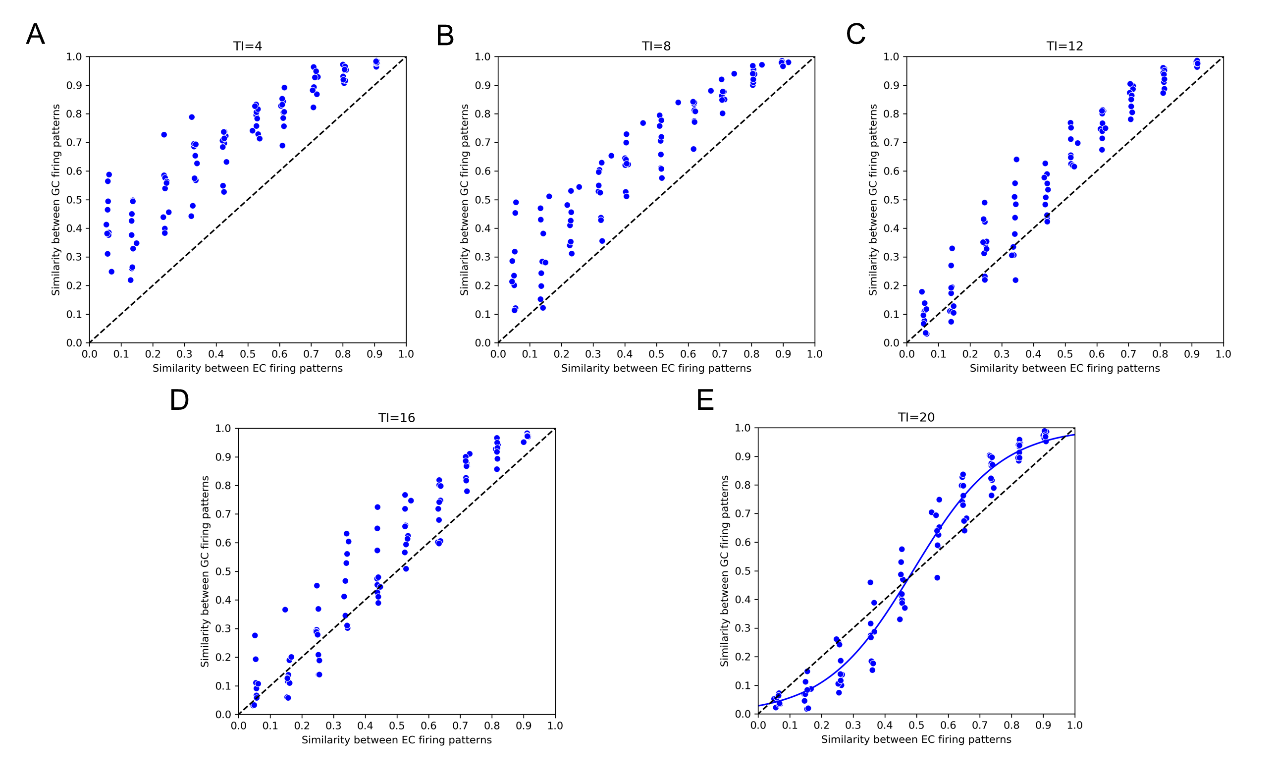


Figure S4. The scatter plots of the relationship between similarity of the firing patterns of EC and the firing patterns of GCs with neurogenesis rate of 3 neurons/s and input time interval of (A) 4 s, (B) 8 s, (C) 12 s, (D) 16 s and (E) 20 s in 200 GCs network with maturation period of 20 s. Each dot represents one concept pair stimuli with similarity between the firing patterns of EC shown along the horizontal-axis and similarity between the firing patterns of GCs shown along the vertical-axis. Sigmoidal relationship can be found between similarity of the firing patterns of EC and that of GCs with input time interval of 20 s. Black dashed lines denote the limit above which pattern separation is performed in the model. TI: time interval.


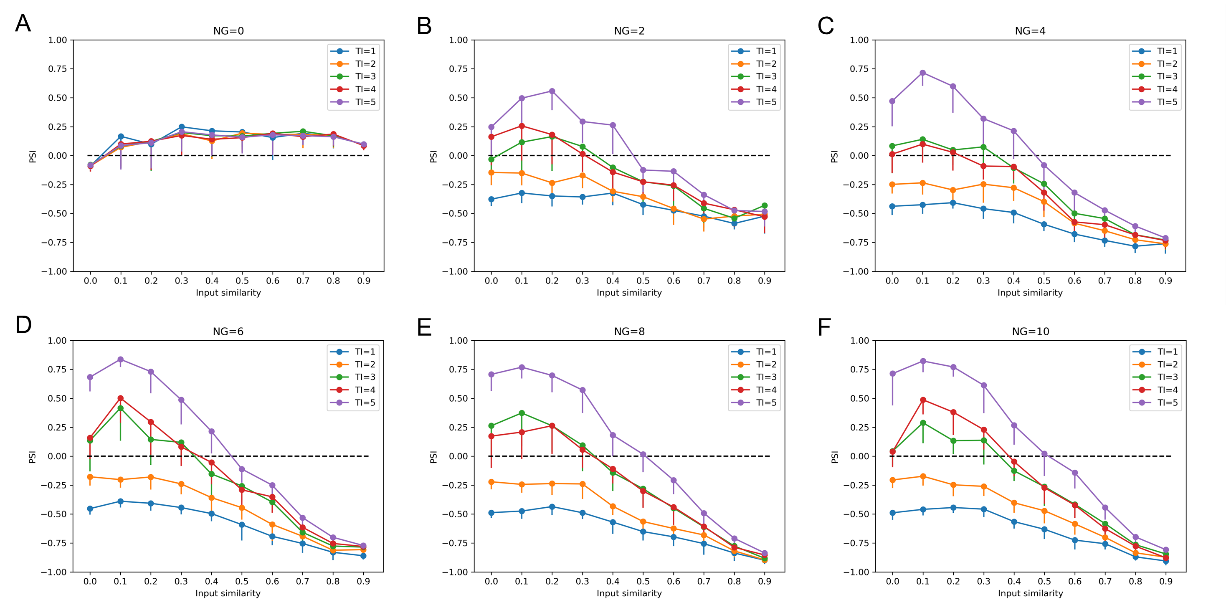


Figure S5. The impact of input similarity and input time interval on concept coding with the neurogenesis rate of (A) 0 neuron/s, (B) 2 neuron/s, (C) 4 neurons/s, (D) 6 neurons/s, (E) 8 neurons/s and (F) 10 neurons/s in the 400 GCs network with maturation period of 5 s, where PSI>0 means pattern separation, PSI<0 indicates pattern integration, and PSI=0 (black dotted line) presents neither pattern separation nor pattern integration. The error bars show standard deviation. NG: neurogenesis rate, TI: time interval.


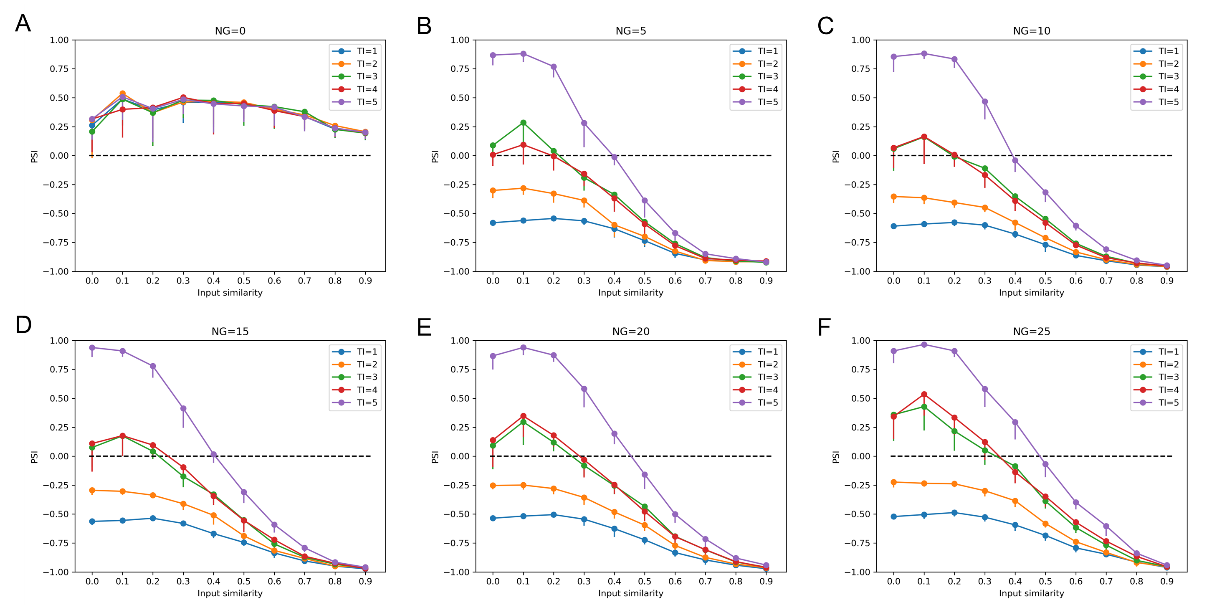


Figure S6. The impact of input similarity and input time interval on concept coding with the neurogenesis rate of (A) 0 neuron/s, (B) 5 neuron/s, (C) 10 neurons/s, (D) 15 neurons/s, (E) 20 neurons/s and (F) 25 neurons/s in the 1,000 GCs network with maturation period of 5 s, where PSI>0 means pattern separation, PSI<0 indicates pattern integration, and PSI=0 (black dotted line) presents neither pattern separation nor pattern integration. The error bars show standard deviation. NG: neurogenesis rate, TI: time interval.


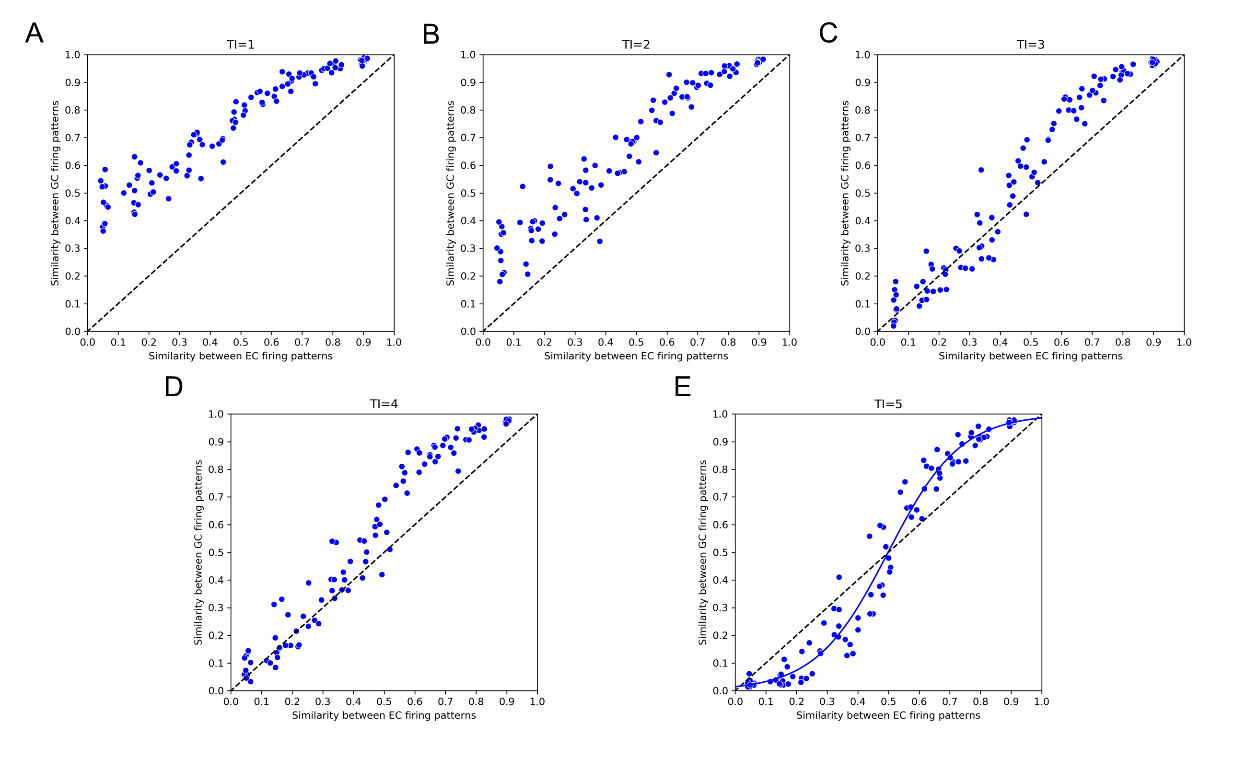


Figure S7. The scatter plots of the relationship between similarity of the firing patterns of EC and the firing patterns of GCs with neurogenesis rate of 4 neurons/s and input time interval of (A) 1 s, (B) 2 s, (C) 3 s, (D) 4 s and (E) 5 s in 400 GCs network with maturation period of 5 s. Each dot represents one concept pair stimuli with similarity between the firing patterns of EC shown along the horizontal-axis and similarity between the firing patterns of GCs shown along the vertical-axis. Sigmoidal relationship can be found between similarity of the firing patterns of EC and that of GCs with input time interval of 5 s. Black dashed lines denote the limit above which pattern separation is performed in the model. TI: time interval.


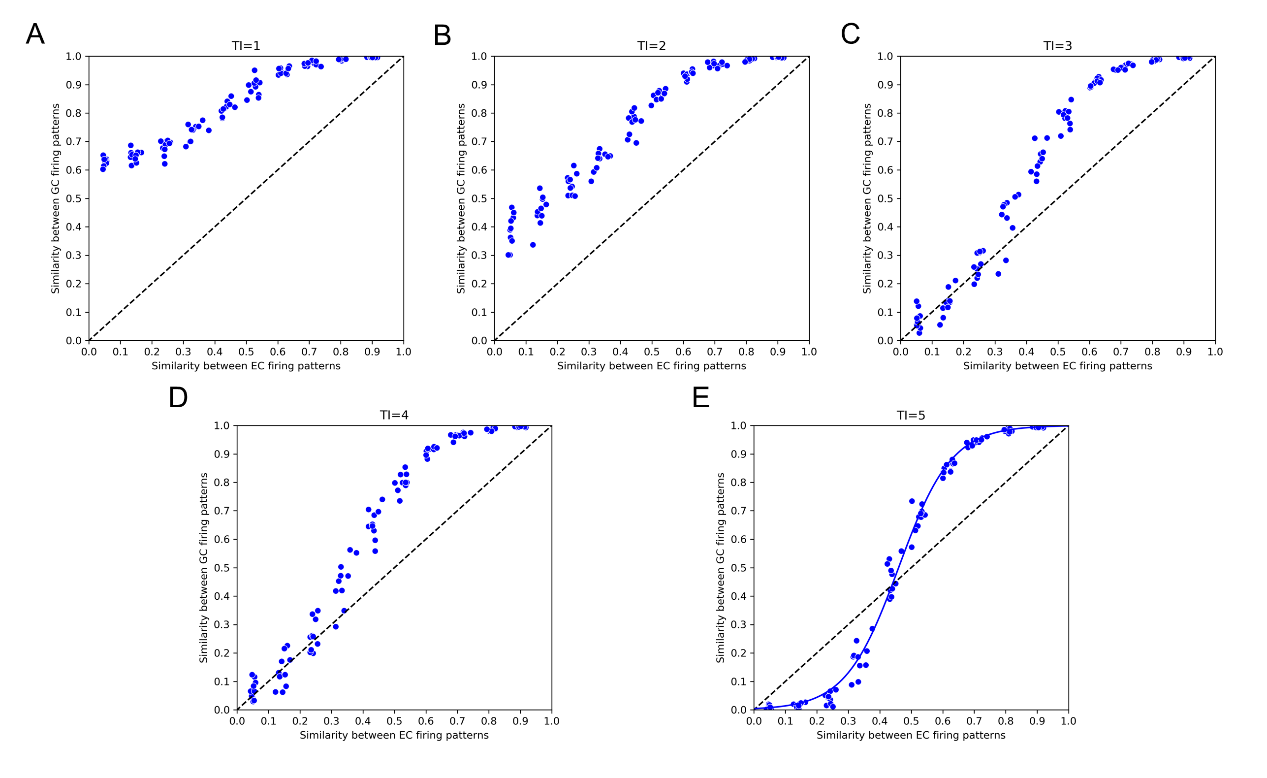


Figure S8. The scatter plots of the relationship between similarity of the firing patterns of EC and the firing patterns of GCs with neurogenesis rate of 10 neurons/s and input time interval of (A) 1 s, (B) 2 s, (C) 3 s, (D) 4 s and (E) 5 s in 1,000 GCs network with maturation period of 5 s. Each dot represents one concept pair stimuli with similarity between the firing patterns of EC shown along the horizontal-axis and similarity between the firing patterns of GCs shown along the vertical-axis. Sigmoidal relationship can be found between similarity of the firing patterns of EC and that of GC with input time interval of 5 s. Black dashed lines denote the limit above which pattern separation is performed in the model. TI: time interval.

# Supplementary Table

Table S1. The contexts and concepts used in simulation.

| **Context** | **Concept** | **Context** | **Concept** |
| --- | --- | --- | --- |
| terrestrial animals | bear | weapons | gun |
|  | buffalo |  | pistol |
|  | cat |  | revolver |
|  | deer |  | shotgun |
| birds | budgie | cigarettes | cigar |
|  | buzzard |  | cigarette |
|  | canary |  | pipe |
|  | crane |  | tobacco |
| body parts | foot | drugs | aspirin |
|  | leg |  | codeine |
|  | thumb |  | ibuprofen |
|  | toe |  | valium |
| vegetables | asparagus | clothing | cloak |
|  | broccoli |  | dress |
|  | cabbage |  | robe |
|  | celery |  | shawl |
| trees | fir | shellfish | clam |
|  | pine |  | mussel |
|  | sycamore |  | oyster |
|  | willow |  | scallop |
| fruits | melon | fish | carp |
|  | nectarine |  | cod |
|  | orange |  | flounder |
|  | peach |  | herring |
| hardware tools | hatchet | paper products | newspaper |
|  | saw |  | pamphlet |
|  | scissors |  | book |
|  | scythe |  | brochure |
